# Effect of Whey Protein Isolate and Concentrate Shakes on Surface and Optical Properties of 3D-Printed Definitive Resins

**DOI:** 10.3390/polym18101166

**Published:** 2026-05-09

**Authors:** Hasan Can Albayrak, Simge Taşın, Artur İsmatullaev

**Affiliations:** Faculty of Dentistry, Cyprus Health and Social Sciences University, Güzelyurt Mersin 10, Türkiye; simge.tasin@kstu.edu.tr (S.T.); artur.ismatullaev@kstu.edu.tr (A.İ.)

**Keywords:** CAD-CAM, 3D printing, definitive resin, surface roughness, color stainability, translucency, whey protein

## Abstract

This in vitro study evaluated the effects of whey protein isolate and concentrate on surface roughness, color stainability, and translucency of 3D-printed definitive resins. Five 3D-printed resins were tested: Alias Dental Crown (AC), Crowntec (CT), Permanent Crown Resin (PC), VarseoSmile Crown^plus^ (VSC), and VarseoSmile TriniQ (VST) (*n* = 30). Each material was subdivided into groups (*n* = 10), and the same specimens were evaluated at baseline, after 3 and 14 days of immersion in distilled water, whey protein isolate, or whey protein concentrate. Surface roughness (Ra) was measured with a contact profilometer, while color stainability (Δ*E*_00_) and translucency (Δ*RTP*_00_) were assessed using a spectrophotometer. Surface roughness was not significantly affected by solution type, with minor material-specific increases limited to AC and VST (*p* = 0.001). Color stainability differed significantly among solutions (*p* = 0.001) and increased significantly between 3 and 14 days (*p* = 0.001), with whey protein concentrate producing the greatest discoloration. At 14 days, AC demonstrated the highest Δ*E*_00_, followed by CT, while PC, VSC, and VST showed comparable performance. Translucency changes differed significantly among materials (*p* = 0.001), with AC exhibiting the lowest Δ*RTP*_00_ in protein solutions, exceeding the perceptibility threshold but remaining below the acceptability threshold. Whey protein shakes increased color stainability of certain resins, with AC, CT, and VST exceeding the color acceptability threshold (AT_00_ = 1.81), while surface roughness changes were limited and translucency remained within the acceptability threshold for all materials.

## 1. Introduction

The rapid development of digital technologies has significantly influenced restorative dentistry and the manufacturing of dental materials. Additive manufacturing (AM), or 3-dimensional (3D) printing, has emerged as an alternative to subtractive computer-aided design and computer-aided manufacturing systems, offering reduced material waste, lower production costs, and the ability to fabricate complex geometries [1,2]. Among the different AM techniques, stereolithography (SLA), digital light processing (DLP), and masked stereolithography (MSLA) rely on photopolymerization to build objects layer by layer [3]. In prosthodontics, 3D-printing has been applied to interim restorations, long-term provisional prostheses, and removable complete or partial dentures [1,4]. Although early generations of 3D-printed polymers exhibited limitations in strength, esthetics, and biological performance [5], recent developments have demonstrated improvements in flexural strength [6,7], wear resistance [8,9], and optical stability [7,10], suggesting their suitability for long-term restorative applications. Nevertheless, their performance remains material-dependent, warranting continued evaluation of their optical and mechanical behavior [11].

The clinical success of definitive restorations is influenced by several factors, including surface characteristics and optical properties such as stain resistance and translucency. Surface roughness is considered one of the factors that may affect the long-term performance of restorations, as it has been associated with stain retention, plaque accumulation, and esthetic longevity [12,13,14,15,16]. Roughness may be influenced by manufacturing methods, subsequent processing, polishing protocols, and aging conditions [13,17]. Immersion of restorative materials in various solutions is a commonly used method to evaluate their behavior under different chemical conditions. Previous studies reported that exposure to different beverages can affect the surface quality and color stainability of resin-based materials [18,19,20,21]. Optical properties, including color stainability and translucency, are also regarded as relevant factors in the clinical performance of definitive restorations [22]. These properties are influenced by factors such as water sorption [23,24], filler characteristics [25], photoinitiator systems [26], thickness, and shade [27,28,29,30,31,32]. Color differences are calculated using the CIEDE2000 formula, which correlates better with visual perception [33], whereas translucency is assessed by comparing color measurements obtained against black and white backgrounds [17,20,30,31,34].

The global sports supplement market has expanded rapidly due to increased health awareness and advances in food technology [35]. Whey protein shakes are widely consumed, particularly among athletes and physically active individuals [36]. Whey protein is a milk-derived protein fraction that consists primarily of β-lactoglobulin, α-lactalbumin, bovine serum albumin, immunoglobulins, and other minor bioactive proteins [37,38]. Commercial whey protein supplements are primarily available in concentrate and isolate forms, which differ in their degree of purification and residual non-protein content [38,39]. In general, isolates contain a higher proportion of protein, whereas concentrates retain greater amounts of lactose, fat, minerals, and suspended solids [38,39]. In addition, commercially available whey protein beverages may include flavoring agents, sweeteners, colorants, stabilizers, and acidifying components, which contribute to their staining potential and slightly acidic pH [37,38]. Their viscous consistency may prolong surface contact time and facilitate protein adsorption onto resin-based materials, potentially increasing erosive and staining effects [40,41]. Previous studies have demonstrated adverse effects of protein shakes on the color and surface properties of resin-based materials [40,42,43,44], and clinically perceptible discoloration has also been reported in zirconia [45,46]. The effect of these beverages on 3D-printed definitive restorative resins has not yet been investigated. Therefore, the present study aimed to evaluate the effects of whey protein isolate and concentrate shakes on surface roughness, color stainability, and translucency of 3D-printed definitive restorative materials. The null hypotheses were that immersion in whey protein isolate and concentrate shakes would not affect (a) surface roughness, (b) color stainability, or (c) translucency of the tested 3D-printed definitive resins.

## 2. Materials and Methods

Information regarding each evaluated material is shown in Table 1. The sample size was calculated using G*Power software (version 3.1; Heinrich-Heine-Universität Düsseldorf, Düsseldorf, Germany). An F-test (ANOVA: fixed effects, omnibus, one-way) was used for the sample size calculation. The effect size (f) was set at 0.38 based on data derived from a previous study with a similar methodology [4]. The significance level (α) was set at 0.05, and the statistical power (1 − β) was set at 0.80. Based on these parameters and the study design, a minimum of 10 specimens per group was determined to be sufficient to achieve the desired statistical power, with an actual power of 0.839.

A disc-shaped specimen (Ø10 × 2 mm) was designed using the FreeCAD 0.19 open-source software and exported as a standard tessellation language (STL) file. A total of 150 specimens were fabricated from five definitive 3D-printed materials: Alias Dental Crown (AC), Crowntec (CT), Permanent Crown Resin (PC), VarseoSmile Crown^plus^ (VSC), and VarseoSmile TriniQ (VST) according to the manufacturers’ instructions.

All materials were printed at a 0-degree build angle with a 50 µm layer thickness according to manufacturers’ instructions. PC was fabricated using an SLA system with a 405 nm laser source, whereas the remaining materials were printed using DLP or MSLA systems with 405 nm ultraviolet light-emitting diode polymerization. Detailed printing and post-curing parameters are provided in Table 2. Both sides of each specimen were polished manually by a single investigator under running water with controlled finger pressure, with each silicon carbide abrasive paper applied for 20 s. Polishing was performed sequentially using 600-, 800-, 1000-, 1200-, 1500-, and 2000-grit papers (NIKON, Shenzhen Sun Abrasives IMP. & EXP. Corp., Shenzhen, China) to ensure standardization and obtain a uniform surface finish. The final thickness (2 ± 0.02 mm) was verified with a digital caliper (Digimatic CD-15DCX, Mitutoyo Corp., Kawasaki, Japan; resolution 0.01 mm, accuracy ±0.02 mm). Subsequently, all specimens were cleaned in an unheated ultrasonic bath containing distilled water for 10 min to remove polishing debris and potential surface contaminants. The specimens were then stored in distilled water at 37 °C for 24 h. Before baseline measurements, each specimen was removed from distilled water and gently air-dried to eliminate excess surface moisture, ensuring standardized testing conditions.

Baseline surface roughness (Ra_0_) was measured using a contact profilometer (MarSurf PS10; Mahr GmbH, Göttingen, Germany; cutoff: 0.8 mm, speed: 0.5 mm/s) equipped with a diamond stylus tip with a radius of 2 µm and a measuring force of 0.7 mN, according to the manufacturer’s specifications. Each specimen was positioned in a fixed holder to ensure measurement stability. Surface roughness was measured from three different locations of the same surface by rotating the specimen by 90° between consecutive measurements to account for surface heterogeneity. The mean Ra value was calculated from these three measurements. Specimens from each material were randomly allocated into three subgroups (*n* = 10), according to the immersion solution: two staining solutions and distilled water. Randomization was performed using a computer-generated number sequence (Microsoft Excel; Microsoft Corp., Redmond, WA, USA). Baseline color parameters (L*, a*, b*) were obtained before immersion using a digital spectrophotometer (VITA Easyshade; Vita Zahnfabrik, Bad Säckingen, Germany). Measurements were performed in a neutral gray-coated box (35 × 35 × 70 mm) against a gray background for color evaluation, and black and white backgrounds for translucency assessment, under D65 illumination with a CIE 2-degree observer. The spectrophotometer (5 mm aperture; accuracy ≈ 93.75%; repeatability 0.81–0.93 CIELAB units) was recalibrated before each measurement, and measurements were performed by a single investigator. Each measurement was repeated 3 times in Tooth Single mode, and mean values were calculated. Specimens were positioned in a custom mold with the probe perpendicular to the surface (90°) during color measurements. After baseline measurements, specimens were fixed in a fabric mesh holder to ensure upright positioning and standardized staining of the measured surfaces.

After baseline measurements, the specimens were immersed in three solutions: 250 mL of distilled water (control), 250 mL of whey protein isolate (Multipower Whey Isolate 100; Multipower Sportsfood GmbH, Hamburg, Germany), or 250 mL of whey protein concentrate (XPRO Concentrate Whey Protein; XPRO Nutrition, Alicante, Spain), and the specimens were evaluated at 3 and 14 days following immersion. Whey protein shakes were prepared according to the manufacturers’ instructions by dissolving 30 g of powder in 250 mL of distilled water at room temperature. Before immersion, the pH values of the whey protein isolate and whey protein concentrate solutions were measured as 5.7 and 5.3, respectively, using a benchtop pH/mV meter (BANTE INSTRUMENTS 210; Bante Instruments Inc., Shanghai, China). According to manufacturer-reported ingredient information, the whey protein isolate (Multipower Whey Isolate 100) contained instantized whey protein isolate (94%), cocoa powder (4%), emulsifier (soya lecithin), flavoring agents, and non-nutritive sweeteners (sodium cyclamate, sodium saccharin, and acesulfame-K). The whey protein concentrate (XPRO Concentrate Whey Protein) contained whey protein concentrate (WPC80) as the primary ingredient, along with flavoring agents (chocolate and white chocolate flavorings), a colorant (caramel color), emulsifier (sunflower lecithin), a thickening agent (guar gum), cocoa, non-nutritive sweeteners (sucralose and acesulfame-K), and an anti-caking agent (silicon dioxide).

Specimens were stored at room temperature in a dark environment, and solutions were continuously stirred at 500 rpm using a magnetic stirrer (Sagrado, İzmir, Türkiye). Solutions were refreshed daily. After each immersion period, specimens were rinsed under distilled water for 5 min and dried with tissue paper before subsequent measurements. After each immersion period, surface roughness was measured first, followed by color and translucency assessments under the same conditions as baseline. Changes in surface roughness were calculated relative to baseline values (ΔRa). The CIEDE2000 (Δ*E*_00_) color difference was calculated against a neutral gray background (L* = 95.4; a* = 0.3; b* = 2.2) using the following formula [47,48]:

$$∆E_{00}=\sqrt{{\left(\frac{∆L*}{K_{L}S_{L}}\right)}^{2}+\;{\left(\frac{∆C*}{K_{C}S_{C}}\right)}^{2}+{\left(\frac{∆H*}{K_{H}S_{H}}\right)}^{2}+R_{T}\left(\frac{∆C*}{K_{C}S_{C}}\right)\;\left(\frac{∆H*}{K_{H}S_{H}}\right)}$$

where constants *K_L_*, *K_C_*, and *K_H_* were set to 1. For Δ*E*_00_ interpretation, the 50% color perceptibility (PT_00_ = 0.81) and acceptability (AT_00_ = 1.81) thresholds were referenced [47]. The relative translucency parameter (*RTP*_00_) was calculated using the CIEDE2000 color difference formula, based on color differences between black (L* = 2.3; a* = 0.7; b* = 1.8) and white (L* = 92.4; a* = 0.9; b* = 1.5) backgrounds. The formula used for this calculation is as follows [48,49]:

$$RTP_{00}=\sqrt{{\left(\frac{L_{B}^{\prime }-L_{W}^{\prime }}{K_{L}S_{L}}\right)}^{2}+{\left(\frac{C_{B}^{\prime }-C_{W}^{\prime }}{K_{C}S_{C}}\right)}^{2}+{\left(\frac{H_{B}^{\prime }-H_{W}^{\prime }}{K_{H}S_{H}}\right)}^{2}+R_{T}\left(\frac{C_{B}^{\prime }-C_{W}^{\prime }}{K_{C}S_{C}}\right)\left(\frac{H_{B}^{\prime }-H_{W}^{\prime }}{K_{H}S_{H}}\right)}$$

where subscript B denotes the measurements obtained over a black background, while the subscript W denotes those obtained over a white background for each of the lightness (*L*′), chroma (*C*′), and hue (*H*′) parameters. For color difference calculations, the parameters *K_L_*, *K_C_*, and *K_H_* were set to 1. *RTP*_00_ values were measured at baseline, after 3 and 14 days. Changes in translucency were calculated relative to baseline values and expressed as Δ*RTP*_00_. Translucency differences were evaluated according to the 50% perceptibility (TPT_00_ = 0.62) and acceptability (TAT_00_ = 2.62) thresholds reported in the literature [50].

Statistical analyses were performed using NCSS 2007 (Number Cruncher Statistical System, LLC, Kaysville, UT, USA). Descriptive statistics (mean, standard deviation, median, minimum, and maximum) were calculated. Normality of the data was assessed using the Shapiro–Wilk test. Intergroup comparisons were performed using the Kruskal–Wallis test followed by Dunn’s post hoc test. Intragroup comparisons over time were conducted as two separate paired analyses: baseline versus 3-day measurements, and baseline versus 14-day measurements. Each interval was analyzed independently using the Wilcoxon signed-rank test, allowing direct assessment of the effect of each immersion period relative to the pre-immersion condition. Statistical significance was set at *p* < 0.05.

## 3. Results

Baseline values of surface roughness, color parameters, and translucency are summarized in Table 3. Significant differences among materials were observed for baseline surface roughness (Ra_0_, *p* = 0.002), color (*L*_0_*, *a*_0_*, *b*_0_*, *p* = 0.001), and relative translucency parameter (*RTP*_00_, *p* < 0.001). For Ra_0_, VSC showed significantly lower values than all other materials, whereas no significant differences were found among AC, CT, PC, and VST. For *L*_0_ and *a*_0_, all materials differed significantly from one another, except PC and VST for *L*_0_, which showed comparable values. For *b*_0_, VSC and VST were statistically comparable, whereas all other pairwise comparisons showed significant differences. For *RTP*_00_, all materials differed significantly from one another, except PC and VSC, which showed comparable values.

Median, minimum, and maximum change in surface roughness (ΔRa) values after 3 and 14 days are presented in Table 4. At 3 days, the Kruskal–Wallis test showed that in the whey protein isolate, AC and VST exhibited significantly higher ΔRa values than the other materials (*p* = 0.001), with no significant difference between them (*p* = 0.940). After 14 days, AC in whey protein isolate and VST in whey protein concentrate demonstrated significantly greater ΔRa values than the remaining materials (*p* = 0.001). Solution type did not significantly influence ΔRa at any time interval (*p* > 0.05). A significant time-dependent effect was observed only for VST (*p* = 0.005).

**Figure 1:**
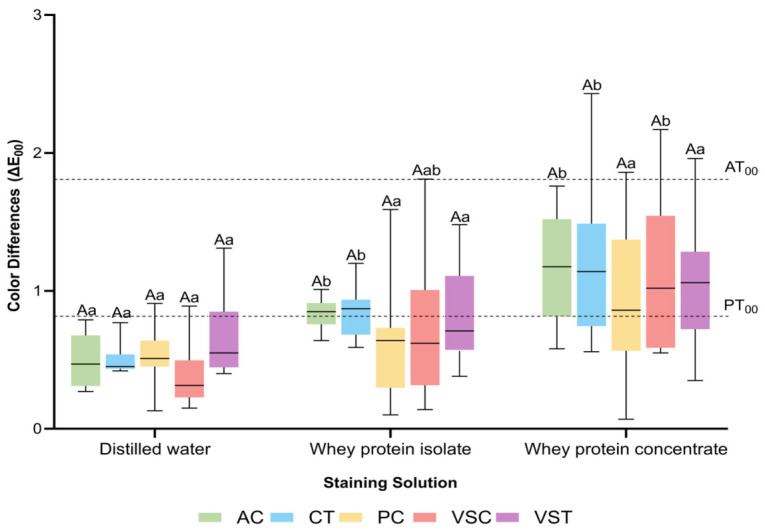
Color difference (CIEDE2000) values of the tested groups at 3 days. AC: Alias Dental Crown; CT: Crowntec; PC: Permanent Crown Resin; VSC: VarseoSmile Crown^plus^; VST: VarseoSmile TriniQ. Different uppercase letters denote statistically significant differences among materials in the same solution. Different lowercase letters denote statistically significant differences among solutions of the same material (*p* < 0.05). The lower and upper dotted lines represent the perceptibility threshold (PT_00_) and acceptability threshold (AT_00_), respectively.

**Figure 2:**
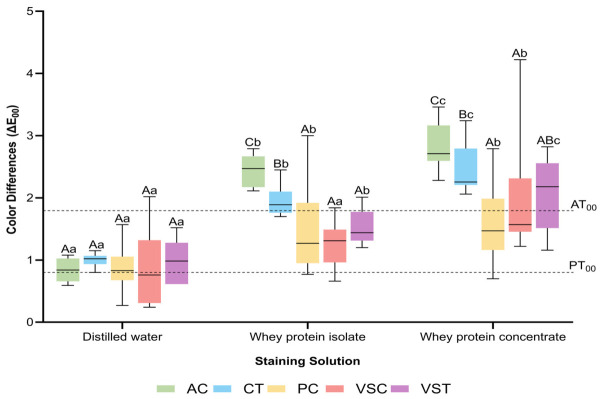
Color difference (CIEDE2000) values of the tested groups at 14 days. AC: Alias Dental Crown; CT: Crowntec; PC: Permanent Crown Resin; VSC: VarseoSmile Crown^plus^; VST: VarseoSmile TriniQ. Different uppercase letters denote statistically significant differences among materials in the same solution. Different lowercase letters denote statistically significant differences among solutions of the same material (*p* < 0.05). The lower and upper dotted lines represent the perceptibility threshold (PT_00_) and acceptability threshold (AT_00_), respectively.

Median Δ*E*_00_ values at 3 and 14 days are shown in Figure 1 and Figure 2. Considering the predefined color perceptibility (PT_00_ = 0.81) and acceptability (AT_00_ = 1.81) thresholds, all materials remained below the acceptability threshold at 3 days. At 3 days, no significant differences among materials were observed within any solution (distilled water: *p* = 0.104; whey protein isolate: *p* = 0.079; whey protein concentrate: *p* = 0.882) (Figure 1). However, the perceptibility threshold was already exceeded by AC (median = 0.85) and CT (median = 0.87) in whey protein isolate, and by all materials in whey protein concentrate, with medians ranging from 0.86 (PC) to 1.18 (AC). Within each material, significant solution-dependent differences were observed for AC, CT (*p* = 0.001), and VSC (*p* = 0.005). For AC and CT, whey protein shakes produced higher Δ*E*_00_ values than distilled water (*p* = 0.001), with no significant difference between the two protein shakes (*p* = 0.082 and *p* = 0.130, respectively). For VSC, whey protein concentrate produced significantly higher values than distilled water (*p* ≤ 0.05), whereas no significant difference was found between whey protein shakes (*p* = 0.096).

At 14 days, significant differences among materials were observed in whey protein isolate and concentrate (*p* = 0.001) (Figure 2). In whey protein isolate, AC demonstrated the highest Δ*E*_00_ (median = 2.47), exceeding the acceptability threshold (AT_00_ = 1.81), followed by CT (median = 1.89), also above the threshold. PC (median = 1.27), VST (median = 1.44), and VSC (median = 1.31) remained below the acceptability threshold in whey protein isolate. In whey protein concentrate, AC again showed the highest median Δ*E*_00_ (2.71), followed by CT (2.26) and VST (2.18), all exceeding the acceptability threshold; PC (median = 1.47) and VSC (median = 1.57) remained below the threshold. In distilled water, all materials remained below the acceptability threshold at 14 days, with medians ranging from 0.76 (VSC) to 1.02 (CT). A significant time-dependent increase in Δ*E*_00_ between 3 and 14 days was observed for AC, CT, PC, and VSC across all solutions (*p* ≤ 0.05), and for VST in protein solutions (*p* ≤ 0.05), indicating progressive color change with extended immersion.

Median, minimum, and maximum Δ*RTP*_00_ values are presented in Table 5. Considering the predefined translucency perceptibility (TPT_00_ = 0.62) and acceptability (TAT_00_ = 2.62) thresholds, none of the materials exceeded the acceptability threshold under any condition at either time point. At 3 days, all Δ*RTP*_00_ median values remained below the perceptibility threshold, indicating clinically imperceptible changes in translucency across all materials and solutions at this time point. In distilled water, translucency changes were comparable among materials (*p* > 0.05), except for PC, which exhibited a small but statistically significant decrease (median = −0.19; *p* = 0.001), although still below the perceptibility threshold. In whey protein isolate, AC demonstrated the lowest median Δ*RTP*_00_ (−0.22; *p* = 0.001), while the remaining materials showed similar distributions. In whey protein concentrate, AC and VST exhibited lower median values (−0.22 and −0.26, respectively; *p* = 0.001), without a statistically significant difference between them (*p* = 0.821); however, all values remained within clinically imperceptible limits.

At 14 days, statistically significant differences among materials were observed in all solutions (*p* = 0.001). In distilled water, AC and CT showed lower median Δ*RTP*_00_ values (−0.06 and −0.09, respectively) compared to VSC (0.11; *p* = 0.001), and these differences remained below the perceptibility threshold (TPT_00_ = 0.62). In whey protein isolate, AC exhibited the lowest median value (−0.68), exceeding the perceptibility threshold in absolute magnitude, whereas VST showed the highest value (0.01; *p* = 0.001). In whey protein concentrate, AC again demonstrated the lowest median Δ*RTP*_00_ (−0.77; *p* = 0.001), also exceeding the perceptibility threshold in absolute magnitude, while the other materials showed comparable median changes that remained below this threshold. Although AC exceeded the perceptibility threshold in both protein solutions at 14 days, all Δ*RTP*_00_ values remained below the acceptability threshold (TAT_00_ = 2.62), indicating that the observed translucency changes were perceptible but not clinically unacceptable.

**Figure 3:**
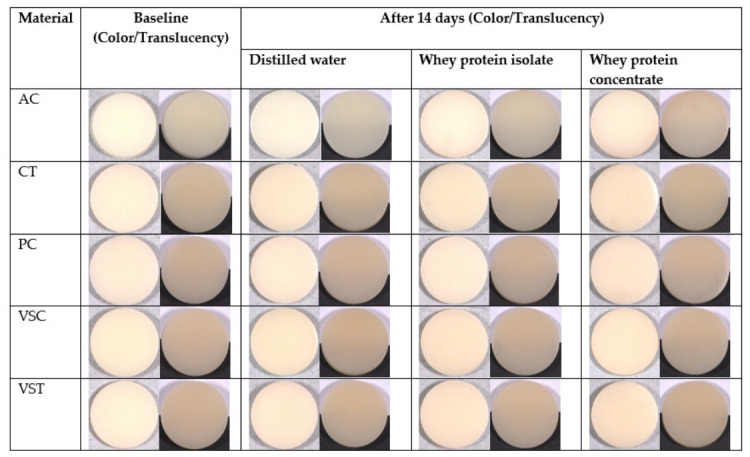
Macroscopic photographs of the tested 3D-printed resins at baseline and after 14 days of immersion in distilled water, whey protein isolate, and whey protein concentrate. A representative specimen from each material group is presented. AC: Alias Dental Crown; CT: Crowntec; PC: Permanent Crown Resin; VSC: VarseoSmile Crown^plus^; VST: VarseoSmile TriniQ.

Standardized macroscopic photographs of the specimens at baseline and after 14 days of immersion are presented in Figure 3. 

The images allow side-by-side visual comparison of color changes among the tested materials and provide qualitative assessment of translucency using white and black backgrounds.

## 4. Discussion

Based on these results, the first null hypothesis that whey protein shakes would not affect surface roughness was accepted, as solution type did not significantly influence ΔRa at any time interval. Although minor material-specific changes were observed for AC and VST, these alterations reflected differences among materials rather than an effect attributable to the immersion solutions themselves. The second null hypothesis stating that whey protein shakes would not affect the color stainability of the tested materials was rejected, as significant differences in Δ*E*_00_ were observed among materials and solutions. The third null hypothesis regarding translucency was rejected, as immersion in protein shakes resulted in a significant reduction in relative translucency compared with distilled water.

Previous studies have primarily compared 3D-printed resins with conventional restorative materials such as resin composites and ceramics [4,9,14,19]. However, with the increasing clinical use of 3D-printed materials indicated for definitive restorations, evaluating these materials as a distinct category is warranted [11]. Accordingly, the present study focused on 3D-printed definitive resins to reduce variability in manufacturing technique and composition.

The specimen thickness was standardized at 2 mm to reduce geometry-related variability and to enable reliable comparison of optical outcomes among materials, since color and translucency are well established as thickness-dependent properties of dental restorative materials [51]. Although previous studies have evaluated the influence of different thicknesses on optical behavior, the present study did not aim to investigate thickness as a variable [51,52]. Therefore, a single standardized thickness was selected to eliminate its potential confounding effect and to allow a more direct comparison of changes related to the tested materials and immersion solutions. In addition, similar specimen dimensions have been used in previous in vitro studies evaluating color stability and surface properties of resin-based materials [4].

Aging of restorative materials is commonly simulated using beverages with established staining potential, such as coffee, tea, and red wine [4,10,14,30]. In contrast, whey protein shakes have rarely been investigated despite their increasing consumption [36]. In the present study, a continuous immersion protocol was used to represent an accelerated cumulative exposure to whey protein beverages. Accordingly, the immersion periods of 3 and 14 days were defined as short- and long-term cumulative exposure conditions, which, based on a theoretical calculation assuming once-daily consumption of approximately 10 min [44], correspond approximately to 1 and 5 years of cumulative beverage exposure, respectively.

Significant baseline differences were observed among the tested materials in surface roughness, color parameters, and translucency, indicating material-dependent initial surface and optical characteristics prior to immersion. These differences may be related to variations in resin composition, including differences in monomer systems, filler content, and photoinitiator chemistry, which have been shown to influence light scattering, light transmission, and surface characteristics in resin-based materials [25,26,27,28]. In addition, differences in printing technology and post-curing protocols may influence polymerization efficiency and microstructural development, thereby potentially contributing to variations in baseline optical and surface properties [26,31]. Furthermore, shade and specimen thickness have also been reported to affect optical outcomes, even under standardized experimental conditions [27,28,29,30,31,32].

In the present study, surface roughness exhibited material-dependent changes, with AC and VST showing greater susceptibility than PC, VSC, and CT. Although statistically significant differences were observed, the absolute ΔRa values remained below the commonly accepted threshold of 0.2 µm for plaque accumulation and well below surface roughness level associated with clinical detectability, which has been reported to be approximately 10 µm [4,14,19]. Previous studies have reported significant roughness increases for PC, VSC, and CT after immersion in coffee and tea [10,14] as well as after brushing and thermocycling [15,16]. In contrast, the whey protein beverages used in the present study produced less pronounced effects, which may be related to differences in pH, aging conditions, and exposure duration. For AC, which contains a UDMA-based matrix, previous studies have suggested that UDMA-based monomer systems may exhibit higher hydrophilicity and water uptake compared with Bis-EMA-based systems, which may influence surface properties such as roughness [23,24,29]. This behavior may contribute to the greater roughness changes observed for this material under the present conditions. For VST, greater roughness changes were observed compared with VSC despite their broadly similar methacrylate-based formulations, suggesting that factors other than monomer composition may be involved. Although the manufacturer does not clearly report the quantitative filler content of VST, a previous study has indicated a relatively lower inorganic filler content (approximately 20 wt%) for this material, and lower filler content has been associated with a higher proportion of resin matrix, which may increase susceptibility to surface alterations [25]. In addition, it should be considered that the materials were fabricated using different printing technologies and post-processing protocols. Variations in post-curing conditions, including light intensity, exposure time, and temperature, have been reported to influence the degree of conversion and surface properties of 3D-printed resins [28,31]. Accordingly, the higher ΔRa values observed for AC and VST may be related to combined effects of material composition and processing conditions.

Color stainability differed significantly among materials within the tested solutions, and differences among solutions were also observed within individual materials. AC and CT exhibited the highest Δ*E*_00_ values in whey protein shakes at both 3 and 14 days, exceeding the clinical AT_00_ of 1.81 [47]. In contrast, VST exceeded the AT_00_ only after 14 days of immersion in whey protein concentrate, indicating a time-dependent effect. For VST, although the manufacturer does not clearly report the quantitative filler content, a previous study has indicated a relatively lower inorganic filler content (approximately 20 wt%) for this material [25]. Resin-based materials with lower filler content have been reported to exhibit greater color change, which has been attributed to the increased proportion of resin matrix facilitating water sorption and the diffusion of staining agents [53]. Accordingly, the observed discoloration in VST may be related to its relatively lower filler content and corresponding resin-dominated structure. For AC, no previous color stainability data are available; however, its pronounced discoloration may be related to its UDMA- and glycol methacrylate-based matrix, which has been suggested to exhibit higher hydrophilicity and water sorption compared with Bis-EMA-based systems [24]. Previous studies have reported that increased water sorption may increase the susceptibility of resin-based materials to staining by facilitating the diffusion of pigments into the polymer network [23,29]. In the present study, the greater discoloration observed for AC may be related to increased pigment uptake associated with its hydrophilic resin matrix. However, CT has been investigated previously, with some studies reporting color changes above the acceptability threshold after coffee immersion or thermocycling [7,14,19,22], whereas others have shown more favorable performance [15]. Previous studies have suggested that both fabrication technologies, particularly additive manufacturing techniques [54], and post-processing procedures [5,31] may influence the color stability of resin-based materials by altering their microstructure and optical behavior. In the present study, the higher discoloration observed for CT may be related to material-specific fabrication and post-processing conditions. In contrast, PC and VSC showed the lowest Δ*E*_00_ values and remained below the clinical threshold, consistent with reports of acceptable color stability [14,15,34], although outcomes may vary depending on aging conditions and solution characteristics [16,19]. Their lower color stainability may be related to relatively more hydrophobic resin matrices and filler characteristics. Furthermore, differences in printing technology, cleaning procedures, and post-curing protocols have been suggested to influence the degree of polymerization and residual monomer content, thereby possibly contributing to variations in staining susceptibility among materials [5,28,31,54]. Accordingly, the relatively lower discoloration observed for PC and VSC may be related to a combination of material characteristics as well as differences in printing technology, cleaning procedures, and post-curing protocols.

When solutions were compared, whey protein shakes induced significantly greater color change than distilled water. Previous studies have suggested that protein adsorption on material surfaces may promote pellicle formation and pigment retention and may be associated with increased bacterial adhesion and biofilm development [41]. These processes may contribute to pigment retention and accumulation on the material surface, potentially influencing the observed discoloration. Whey protein shakes have previously been reported to cause perceptible staining in resin-based and ceramic materials [43,45,46]. The tested whey protein shakes consisted of commercially available formulations rather than standardized isolate and concentrate compositions; their detailed ingredient profiles are provided in the Materials and Methods section. Both formulations included additional components beyond whey protein, which may have contributed to the observed staining behavior. However, the whey protein concentrate generally exhibited a greater staining effect compared to the isolate formulation. Notably, the concentrate formulation additionally contained a caramel-based colorant and sucralose, whereas the isolate formulation contained sodium cyclamate and saccharin without a declared colorant, which may partly account for the greater discoloration observed with the concentrate. Furthermore, a significant increase in color change was observed between 3 and 14 days, indicating a time-dependent effect on color stainability (*p* = 0.005), which may be related to cumulative pigment adsorption and progressive interactions between staining agents and the resin matrix.

Translucency changes remained within the acceptability threshold (TAT_00_ = 2.62) for all materials at both time points. However, AC exceeded the perceptibility threshold (TPT_00_ = 0.62) in absolute magnitude after 14 days of immersion in whey protein isolate (−0.68) and concentrate (−0.77), indicating perceptible but clinically acceptable translucency changes for this material. The remaining materials exhibited comparable Δ*RTP*_00_ values that remained below the perceptibility threshold. Previous studies have suggested that curing parameters, including light source, exposure time, and temperature, as well as hydrothermal challenges such as coffee thermocycling, may influence translucency, although most alterations remain within clinical thresholds [28,31]. Variations in photoinitiator systems have also been suggested to influence polymerization efficiency and network homogeneity, thereby potentially affecting optical behavior and contributing to differences in translucency stability [26]. These differences may be related to material-specific post-polymerization conditions and photoinitiator systems, particularly in AC.

From a clinical perspective, the present findings highlight material-dependent differences in discoloration behavior among 3D-printed definitive resins under accelerated immersion conditions. While certain materials demonstrated greater color change, these observations primarily reflect variations in staining susceptibility under controlled experimental conditions. Specifically, AC, CT, and VST demonstrated greater susceptibility to staining, particularly in whey protein concentrate, whereas PC and VSC showed more favorable color stability under the present conditions. Accordingly, these findings should be interpreted as indicative of relative material performance rather than as direct clinical recommendations.

Limitations of this study include the in vitro design, the use of standardized disc specimens, and the evaluation of a single specimen thickness, shade, and polishing protocol. The effects of tooth substrate and luting agent shade were not assessed. Aging conditions were limited to immersion without thermocycling, mechanical loading, brushing, or ultraviolet exposure. In addition, the experimental model did not simulate the oral environment, as factors such as artificial saliva, salivary pellicle formation, biofilm accumulation, mechanical wear, pH fluctuations, and thermal variations were not incorporated. The chemical composition of the tested whey protein shakes was not characterized beyond manufacturer-reported information. Furthermore, only two commercially available whey protein formulations were evaluated, which may limit the generalizability of the findings to other products with different compositions. The clinical year equivalences attributed to the immersion periods of 3 and 14 days represent approximate theoretical estimates based on assumed daily consumption patterns rather than validated measures of clinical service duration. Although the non-parametric approach was appropriate for the distributional characteristics of the data, formal statistical modeling of the interaction effects among material, solution, and time factors was beyond the scope of the current sample size and represents an avenue for future investigation. In addition, the sample size estimation was based on a one-way ANOVA framework, which corresponds to the primary pairwise comparisons of the present design but does not fully capture its multifactorial and longitudinal structure; the present sample size may therefore have been insufficient to detect higher-order interaction effects. Future studies incorporating more clinically relevant aging conditions and a wider range of materials and formulations are warranted to better define the long-term optical and surface performance of 3D-printed definitive resins.

## 5. Conclusions

Within the limitations of this in vitro study, the tested 3D-printed definitive resins responded differently to immersion in whey protein shakes. Color stainability was the most affected property, with AC, CT, and VST exceeding the color acceptability threshold (AT_00_ = 1.81) after 14 days, and the concentrate formulation producing greater discoloration than the isolate. Surface roughness was not influenced by solution type, and the limited changes observed were confined to AC and VST. Translucency remained within the acceptability threshold for all materials, although AC showed perceptible reductions after 14 days in both protein solutions.

## Figures and Tables

**Table 1:** Materials evaluated.

Material	Code	Composition	Shade/Lot No.	Production Technology	Manufacturer
Alias Dental Crown	AC	30–50 wt% inorganic fillers (0.7 µm glass filler), UDMA, glycol methacrylate, and phosphine oxide	A2/240591	MSLA	Dokuz Kimya (Aydın Türkiye)
Crowntec	CT	Bis-EMA, methacrylate polymer, 4,4′-isopropylidenediphenol, ethoxylated and 2-methylprop-2-enoic acid, methyl benzoylformate, diphenyl (2,4,6-trimethylbenzoyl) phosphine oxide; 30–50 wt% inorganic fillers	A2/E522	DLP	Saremco Dental AG (Rebstein, Switzerland)
Permanent Crown Resin	PC	Esterification products of 4,4′-isopropylidenediphenol, ethoxylated and 2-methylprop-2-enoic acid (50–70 wt%), diphenyl (2,4,6-trimethylbenzoyl) phosphine oxide (<3 wt%); 30–50 wt% inorganic fillers (700 nm)	A2/602187	SLA	Formlabs Inc. (Somerville, MA, USA)
VarseoSmile Crown^plus^	VSC	Esterification products of 4,4′- isopropylidenediphenol, ethoxylated and 2-methylprop-2-enoic acid, silanized dental glass, methyl benzoylformate, diphenyl (2,4,6-trimethylbenzoyl) phosphine oxide; 30–50 wt% inorganic fillers	A2/601028	DLP	BEGO (Bremen, Germany)
VarseoSmile TriniQ	VST	4,4′-isopropylidenediphenol, ethoxylated and 2-methylprop-2-enoic acid, benzene acetic acid, alpha-oxo-methyl ester, diphenyl (2,4,6-trimethylbenzoyl) phosphine oxide	A2/601795	DLP	BEGO (Bremen, Germany)

Bis-EMA: ethoxylated bisphenol A dimethacrylate; DLP: digital light processing; MSLA: masked stereolithography; UDMA: urethane dimethacrylate.

**Table 2:** Printing and post-curing parameters of 3D-printed definitive resin materials.

Material	Printer Model	Cleaning Procedure	Post-Curing Device	Post-Curing Parameters
AC	Photon Mono X	Ultrasonic bath with 99% isopropyl alcohol for 5 min, air-dried	ShapeCure UV	5 min
CT	Asiga Max UV	Manual cleaning with a cloth soaked in 96% ethanol	Otoflash G171	4000 flashes
PC	Form 3B	Ultrasonic bath with 99% isopropyl alcohol for 3 min	Form Cure	Two cycles of 20 min at 60 °C
VSC	Varseo Xs	Ultrasonic bath with reusable ethanol for 3 min followed by fresh ethanol for 2 min (unheated)	Otoflash	Two cycles of 1500 flashes
VST	Asiga Max UV	Ultrasonic bath with reusable ethanol for 3 min followed by fresh ethanol for 2 min (unheated)	Otoflash	Two cycles of 2000 flashes

AC: Alias Dental Crown; CT: Crowntec; PC: Permanent Crown Resin; VSC: VarseoSmile Crown^plus^; VST: VarseoSmile TriniQ.

**Table 3:** Median (minimum/maximum) baseline values of surface roughness (Ra_0_), color parameters (*L*_0_*, *a*_0_*, *b*_0_*), and relative translucency parameter (*RTP*_00_) of the evaluated 3D-printed definitive resins.

Material	Baseline Values Med (Min/Max)				
	Ra_0_	*L*_0_*	*a*_0_*	*b*_0_*	*RTP*_00_
AC	0.153 (0.111/0.184) ^a^	83.93 (83.27/85.2) ^d^	−1.3 (−1.97/−0.87) ^a^	20.07 (18.23/21.20) ^a^	6.65 (6.43/7.05) ^d^
CT	0.167 (0.090/0.191) ^a^	80.98 (80.53/83.07) ^a^	−0.37 (−0.9/−0.2) ^b^	22.59 (22.07/24.90) ^b^	6.25 (5.99/6.56) ^b^
PC	0.150 (0.105/0.192) ^a^	82.33 (81.57/83.07) ^c^	0.7 (0.57/0.7) ^d^	24.77 (24.27/25.40) ^c^	5.04 (4.71/5.64) ^a^
VSC	0.139 (0.106/0.178) ^b^	81.43 (81.07/81.73) ^b^	0.6 (0.5/0.7) ^c^	25.90 (25.63/26.23) ^d^	5.07 (4.69/5.46) ^a^
VST	0.165 (0.126/0.186) ^a^	82.57 (81.87/83.33) ^c^	0.8 (0.7/0.9) ^e^	26.03 (25.63/26.60) ^d^	5.28 (4.81/5.61) ^c^

AC: Alias Dental Crown; CT: Crowntec; PC: Permanent Crown Resin; VSC: VarseoSmile Crown^plus^; VST: VarseoSmile TriniQ; Ra_0_: baseline surface roughness; *L*_0_*: lightness; *a*_0_*: red–green coordinate; *b*_0_*: yellow–blue coordinate; *RTP*_00_: relative translucency parameter. Different superscript letters in the same column indicate statistically significant differences among materials (*p* < 0.05).

**Table 4:** Median (Med), minimum (Min), and maximum (Max) ΔRa values (µm) of tested materials after 3 and 14 days.

Material	Distilled Water Med (Min/Max)		Whey Protein Isolate Med (Min/Max)		Whey Protein Concentrate Med (Min/Max)	
	Day 3	Day 14	Day 3	Day 14	Day 3	Day 14
AC	0.03 (−0.03/0.06) ^Aa^	0.01 (−0.03/0.05) ^Aa^	0.03 (0.01/0.04) ^Aa^	0.04 (0.02/0.06) ^Bb^	0.01 (0.00/0.08) ^Aa^	0.01 (−0.01/0.08) ^Aab^
CT	0.01 (−0.01/0.03) ^Aa^	0.01 (0.00/0.03) ^Aa^	0.00 (−0.01/0.02) ^Ba^	0.00 (0.00/0.02) ^Aa^	0.01 (−0.02/0.02) ^Aa^	0.01 (−0.01/0.05) ^Aa^
PC	0.01 (0.00/0.02) ^Aa^	0.01 (−0.02/0.03) ^Aa^	0.01 (0.00/0.04) ^BCa^	0.02 (−0.01/0.05) ^Aa^	0.01 (−0.03/0.07) ^Aa^	0.02 (−0.01/0.04) ^Aa^
VSC	0.01 (−0.02/0.05) ^Aa^	0.00 (−0.03/0.02) ^Aa^	0.01 (0.00/0.04) ^ABa^	0.01 (0.00/0.04) ^Aa^	0.00 (−0.03/0.03) ^Aa^	0.02 (−0.03/0.04) ^Aa^
VST	0.02 (−0.01/0.07) ^Aa^	0.00 (−0.01/0.08) ^Aa^	0.02 (0.00/0.07) ^ACa^	0.04 (−0.02/0.18) ^ABab^	−0.01 (−0.05/0.06) ^Aa^	0.07 (0.03/0.16) ^Bb^

AC: Alias Dental Crown; CT: Crowntec; PC: Permanent Crown Resin; VSC: VarseoSmile Crown^plus^; VST: VarseoSmile TriniQ. Different uppercase letters denote statistically significant differences among materials in the same solution. Different lowercase letters denote statistically significant differences among solutions of the same material (*p* < 0.05).

**Table 5:** Median (Med), minimum (Min), and maximum (Max) values of relative translucency parameter difference (Δ*RTP*_00_) after 3 and 14 days.

Material	Distilled Water Med (Min/Max)		Whey Protein Isolate Med (Min/Max)		Whey Protein Concentrate Med (Min/Max)	
	Day 3	Day 14	Day 3	Day 14	Day 3	Day 14
AC	−0.07 (−0.29/0.39) ^Aa^	−0.06 (−0.67/0.36) ^Aa^	−0.22 (−0.42/−0.01) ^Ab^	−0.68 (−1.02/−0.45) ^Cb^	−0.22 (−0.35/−0.13) ^Bb^	−0.77 (−0.81/−0.69) ^Bb^
CT	0.13 (−0.2/0.50) ^Aa^	−0.09 (−0.45/0.24) ^ACa^	0.04 (−0.14/0.02) ^Bb^	−0.26 (−0.47/0.02) ^Ba^	0.25 (0.14/0.33) ^Ac^	−0.25 (−0.29/−0.08) ^Aa^
PC	−0.19 (−0.49/0.02) ^Ba^	0.02 (−0.16/0.22) ^ABCa^	−0.14 (−0.56/0.08) ^ACa^	−0.08 (−0.31/−0.00) ^Ba^	0.15 (−0.06/0.35) ^Ab^	−0.11 (−0.17/−0.07) ^Aa^
VSC	0.02 (−0.08/0.32) ^Aa^	0.11 (−0.12/0.34) ^Ba^	−0.03 (−0.25/0.17) ^BCa^	−0.18 (−0.39/−0.01) ^Bb^	0.01 (−0.23/0.29) ^Aa^	−0.23 (−0.44/0.18) ^Ab^
VST	0.14 (−0.28/0.49) ^Aa^	0.23 (−0.39/0.47) ^BCa^	−0.12 (−0.25/0.25) ^ABa^	0.01 (−0.2/0.17) ^Aab^	−0.26 (−0.40/−0.17) ^Bb^	−0.22 (−0.37/0.05) ^Ab^

AC: Alias Dental Crown; CT: Crowntec; PC: Permanent Crown Resin; VSC: VarseoSmile Crown^plus^; VST: VarseoSmile TriniQ. Different uppercase letters denote statistically significant differences among materials in the same solution. Different lowercase letters denote statistically significant differences among solutions of the same material (*p* < 0.05).

## Data Availability

Data are available on request.

## Abbreviations

The following abbreviations are used in this manuscript:

AC	Alias Dental Crown
AT_00_	Color acceptability threshold
Bis-EMA	Ethoxylated bisphenol A dimethacrylate
CAD-CAM	Computer-aided design and computer-aided manufacturing
CT	Crowntec
DLP	Digital light processing
MSLA	Masked stereolithography
PC	Permanent Crown Resin
PT_00_	Color perceptibility threshold
Ra	Surface roughness (arithmetical mean height)
*RTP*_00_	Relative translucency parameter
SLA	Stereolithography
STL	Standard tessellation language
TAT_00_	Translucency acceptability threshold
TPT_00_	Translucency perceptibility threshold
UDMA	Urethane dimethacrylate
VSC	VarseoSmile Crown^plus^
VST	VarseoSmile TriniQ
Δ*E*_00_	Color difference (CIEDE2000)
ΔRa	Change in surface roughness
Δ*RTP*_00_	Change in relative translucency
